# ^11^C-Methionine uptake in meningiomas after stereotactic radiotherapy

**DOI:** 10.1007/s12149-024-01932-6

**Published:** 2024-05-08

**Authors:** Hanne-Rinck Jeltema, Bart R. J. van Dijken, Katalin Tamási, Gea Drost, Mart A. A. M. Heesters, Anouk van der Hoorn, Andor W. J. M. Glaudemans, J. Marc C. van Dijk

**Affiliations:** 1 Department of Neurosurgery, University of Groningen, University Medical Center Groningen, Hanzeplein 1, P.O. Box 30.001, 9700RB Groningen, The Netherlands; 2 Department of Radiology, University of Groningen, University Medical Center Groningen, Groningen, The Netherlands; 3 Department of Epidemiology, University of Groningen, University Medical Center Groningen, Groningen, The Netherlands; 4 Department of Neurology, University of Groningen, University Medical Center Groningen, Groningen, The Netherlands; 5 Department of Radiotherapy, University of Groningen, University Medical Center Groningen, Groningen, The Netherlands; 6 Department of Nuclear Medicine and Molecular Imaging, University of Groningen, University Medical Center Groningen, Groningen, The Netherlands

**Keywords:** Meningioma, ^11^C-Methionine, MET-PET, Stereotactic radiotherapy, Follow-up

## Abstract

**Objective:**

^11^C-Methionine positron emission tomography (MET-PET) is used for stereotactic radiotherapy planning in meningioma patients. The role of MET-PET during subsequent follow-up (FU) is unclear. We analyzed the uptake of ^11^C-Methionine before and after stereotactic radiotherapy (SRT) in patients with a complex meningioma and investigated if there was a difference between patients with progressive disease (PD) and stable disease (SD) during FU.

**Methods:**

This retrospective study investigates 62 MET-PETs in 29 complex meningioma patients. Standardized uptake value (SUV)_max_ and SUV_peak_ tumor-to-normal ratios (T/N-ratios) were calculated, comparing the tumor region with both the mirroring intracranial area and the right frontal gray matter. The difference in ^11^C-Methionine uptake pre- and post-SRT was analyzed, as well as the change in uptake between PD or SD.

**Results:**

Median (IQR) FU duration was 67 months (50.5–91.0). The uptake of ^11^C-Methionine in meningiomas remained increased after SRT. Neither a statistically significant difference between MET-PETs before and after SRT was encountered, nor a significant difference in one of the four T/N-ratios between patients with SD versus PD with median (IQR) SUV_max_ T/N_R front_ 2.65 (2.13–3.68) vs 2.97 (1.55–3.54) [*p* = 0.66]; SUV_max_ T/N_mirror_ 2.92 (2.19–3.71) vs 2.95 (1.74–3.60) [*p* = 0.61]; SUV_peak_ T/N_R front_ 2.35 (1.64–3.40) vs 2.25 (1.44–3.74) [*p* = 0.80]; SUV_peak_ T/N_mirror_ 2.38 (1.91–3.36) vs 2.35 (1.56–3.72) [*p* = 0.95].

**Conclusions:**

Our data do not support use of MET-PET during FU of complex intracranial meningiomas after SRT. MET-PET could not differentiate between progressive or stable disease.

**Supplementary Information:**

The online version contains supplementary material available at 10.1007/s12149-024-01932-6.

## Introduction

Meningiomas are the most encountered intracranial tumors [1]. Meningiomas at the skullbase or in close relation with large vascular structures, e.g., the venous sinuses, are challenging to treat. These patients often undergo multimodal treatment. If possible, surgical resection is an essential part of the treatment. Stereotactic radiotherapy (SRT) is often employed to treat a post-surgical remnant or to prevent further growth of inoperable (rest) lesions. Complex meningioma patients frequently have undergone multiple neurosurgical resections and/or multiple irradiation sessions. Following multimodal treatment, regular MRI follow-up (FU) may be difficult to interpret. Treatment effects (pseudoprogression) and postoperative scar tissue can mimic residual tumor or progression. Also, distinction whether the tumor is invading in bony structures is difficult to judge on MRI. These examples illustrate the need for improvement of FU imaging in these patient [2].

PET imaging has been used for various purposes in the treatment of meningioma. Although FDG-PET is often used for neoplasms in the body, it is not first choice for intracranial neoplasms, because of the high glucose background metabolism in the brain which hinders distinctiveness between normal brain tissue and neoplasm. To overcome this, amino acid tracers are alternatives to be used for PET of intracranial neoplasms [2, 3]. Amino acid PET imaging is used for different types of oncological intracranial disease, e.g., primary brain tumors [4–8], meningiomas [9, 10] and metastasis [11–13]. And it is used for different aspects of the neuro-oncological treatment, e.g., tumor detection, prognostication [14, 15], prediction of tumor grade, optimizing target delineation for radiotherapy [16–18], evaluation of treatment response [19, 20] and surveillance [21]. Regarding the role of PET in neuro-oncology, both Verger et al. and Holzgreve et al. wrote a recent review addressing this topic [22, 23]. Also, there was a semi-recent publication on the topic from the RANO/PET-group focusing on its role in meningioma patients [24] with a recent update of members of the same group [25].

^11^C-Methionine PET (MET-PET) is an example of amino acid metabolism-based PET. Elevated standardized uptake value (SUV) is associated with proliferation [26, 27]. For cell division, DNA replication and biomass generation are needed, which thrive on amino acids as building blocks. Intracellular uptake of amino acid tracers is an active process facilitated by LAT1 and LAT2 transporter. Tumor activity is excellently correlated with an increased uptake of radioactive labeled amino acids. Originally, ^11^C-Methionine was one of the first and most established amino acid radiotracers in neuro-oncology. This tracer has a relatively short half-life and can therefore only be deployed in hospitals that possess an onsite cyclotron. The value of MET-PET during FU in meningioma patients is currently under debate. There is literature advocating its merits [28–30], but a recent small-size prospective pilot study failed to show that combined MRI/MET-PET was superior to MRI-only in detecting disease activity during FU. In the absence of a significant decrease in ^11^C-Methionine uptake many years after SRT, the value of MET-PET during FU of stereotactic irradiated meningiomas remains questionable. However, this pilot study did not contain patients with progressive disease after SRT [31]. Our institution has a longstanding tradition of planning SRT for meningiomas on both MRI and MET-PET, with a relatively large series of pre- and post-SRT MET-PETs. For the current study we aimed to elucidate the uptake pattern of ^11^C-Methionine during FU of complex meningiomas after SRT.

## Materials and methods

### Patient selection

Patients with the following criteria were included: (1) patients with an intracranial meningioma (all WHO grades) on a complex anatomical localization (the skullbase, in proximity of the venous sinuses); (2) SRT targeting on both MRI and MET-PET; (3) one or more post-SRT MET-PETs during FU available for this study. Patients were excluded if the meningioma did not show ^11^C-Methionine uptake, which is very rare. Thirty-six patients met the inclusion criteria. Due to missing MET-PET data (MET-PET scans were occasionally saved in snapshot format, not usable for quantitative measurements anymore), six patients were excluded. Another patient with a MET-PET negative meningioma (both pre-SRT and during FU) was excluded. Therefore, 29 patients were selected for final inclusion, harbouring 30 meningiomas that were treated with SRT based on combined MRI/MET-PET planning.

### Radiotherapy

All included patients were treated with SRT on a linear accelerator (LINAC), either fractionated or with a single shot. For many years, SRT-treatment planning of complex meningiomas was based on both 3D-MRI and MET-PET. At the discretion of the treating physician, MET-PET was subsequently used during FU.

### ^11^C-Methionine PET

The MET-PET scans were acquired on an integrated PET/CT camera system (Biograph mCT 40 or 64 slice PET/CT, Siemens, Knoxville, TN, USA), following EANM procedure guidelines for brain tumor imaging using labeled amino acid analogues [32]. Patients fasted for at least 6 h before the intravenous injection of 200 MBq ^11^C-Methionine. Static PET imaging for 5 min was performed ± 20 min after administration of the tracer. Low-dose CT was performed for attenuation correction and anatomic mapping with 80 kv and 30 mas. The Syngo.via software package (Version VB60, Siemens Healthineers, Forchheim, Germany) was used for imaging analysis. SUVs of the tumor region, the mirroring contralateral side, and the healthy right frontal gray matter were measured. The tumor region of interest (ROI) was captured in an ellipsoid, as well as the contralateral mirroring ROI and healthy right frontal gray matter ROI. The ellipsoid ROI’s were manually set. An example of how the data acquisition was performed is available as supplementary data. Tumor-to-normal (T/N) ratios were calculated. Two types of T/N-ratios were obtained: T/N_mirror_ and T/N_R front_. Two types of SUV were registered: SUV_max_ and SUV_peak_. This resulted in four different T/N-ratios (SUV_max_ T/N_R front,_ SUV_max_ T/N_mirror,_ SUV_peak_ T/N_R front,_ SUV_peak_ T/N_mirror_). This method was designed to make the results more robust and less dependent on type of SUV and protocol to determine the T/N-ratio. If pre-SRT MET-PET data were available for a specific patient, ∆ T/N ratio_preSRT/post-SRT_ and the percentage of uptake increase/decrease was determined during FU.

### Follow up

All patients and FU imaging were discussed in a multidisciplinary tumor board. PD was defined as the decision by the tumorboard that the FU imaging (MRI and/or MET-PET) or clinical course necessitated a next line in treatment, which could be (re)operation, (re)irradiation, systemic treatment, treatment in a clinical trial or best supportive care.

### Statistical analysis

Descriptive statistics were used to describe the patient population. Scatterplots were created to display ^11^C-Methionine uptake over time and Spearman’s *ρ* for non-parametric data was calculated to assess the correlation between variables. Differences in T/N ratio pre- and post-SRT and between patients with SD and PD were assessed using the Mann–Whitney *U* test with effect size. The SPSS software package was used (28.0 IBM, Chicago, Illinois). A two-sided *p* < 0.05 was considered significant.

### Ethical considerations

The study was approved by the local ethical committee. The need to obtain informed consent was waived. The study was registered as an audit with institutional approval (register number *202100283*). No identifiable data is presented. The protocol is in line with the Declaration of Helsinki and its later amendments.

## Results

### Patients

**Fig. 1:**
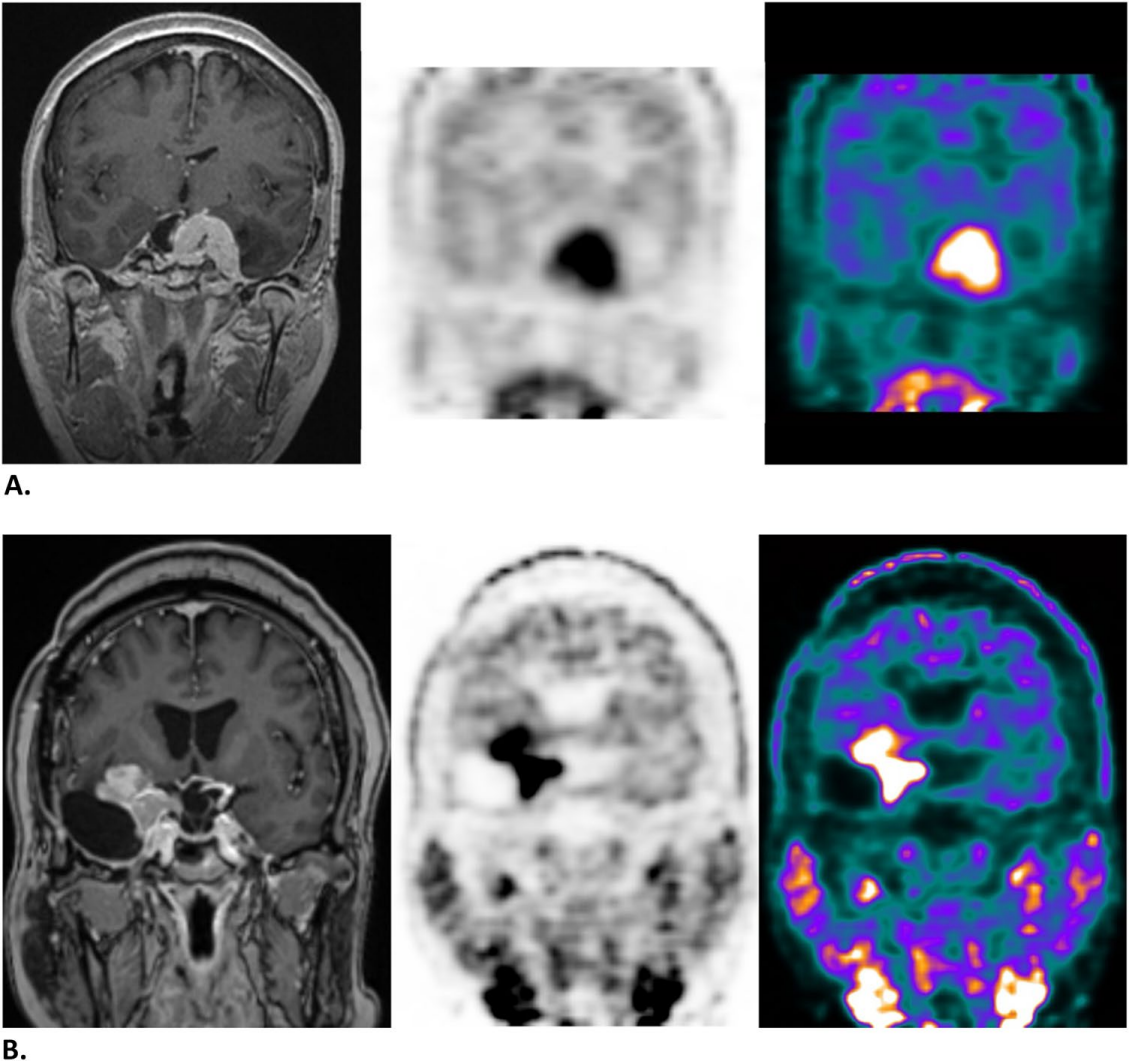
**A** Coronal T1 gadolinium-enhanced MRI and MET-PET scan of a patient with a meningioma in the left cavernous sinus with stable disease 49 months after SRT. **B** Coronal T1 gadolinium-enhanced MRI and MET-PET scan of a patient with a right sided sphenoidal meningioma with progressive disease 97 months after SRT and with a history of previous neurosurgical resections before radiotherapy. *MRI* magnetic resonance imaging, *MET-PET* ^11^C-Methionine positron emission tomography, *SRT* stereotactic radiotherapy

Baseline patient- and tumor characteristics are depicted in Table 1. A total of 29 patients was included, with 30 meningiomas undergoing SRT based on both MRI and MET-PET based planning. The median (IQR) FU duration of the included patients was 67.0 months (50.5–91). Unfortunately some of the older meningioma MET-PET scans were saved in snapshot format and could not be used for quantitative measurements anymore. 62 MET-PET scans were accessable (15 pre-SRT and 47 post-SRT), with a median (IQR) of 2 (1.0–3.0) scans per patient. The median (IQR) interval between SRT and MET-PET was 49.3 months (31.5–84.6). The median (IQR) age at SRT was 50.9 years (45.6–62.6). Most patients (*n* = 21) received 54Gy in 30 fractions. Six patients were treated with a single shot of 14Gy or 20Gy. Three meningiomas were treated with a slightly adjusted radiotherapy scheme (29 × 1.8Gy, 30 × 2.0Gy, or 6 × 5Gy) based on WHO grade or previous treatment. Four patients had PD after SRT, with a total of six “progressive disease” MET-PET scans during FU in this patient group. Characteristics of the treatment and histopathology of patients with PD are given in Table 2. Follow-up MRI and MET-PET of a SD-patient versus a PD-patient are shown in Fig. 1*.* No significant relation between tumor location and ^11^C-Methionine uptake was found.

**Table 1:** Patient and tumor baseline characteristics

*n* = 29 patients with *n* = 30 MET-PET and MRI based SRT treated meningiomas	
Median age (IQR) at SRT	50.9 (45.6–62.6)
Median age (IQR) at last moment of follow-up	58.3 (48.9–68.4)
Sex, *n* (%)	
Female	22 (75.9%)
Male	7 (24.1%)
Multiple meningiomas, *n* (%)	
No	23 (79.3%)
Yes	6 (20.7%)
Location of meningioma, *n* (%)	
Sphenoidal	2 (6.7%)
Frontal skullbase	1 (3.3%)
Clivus	1 (3.3%)
Parasagittal	4 (13.3%)
Cavernous sinus	8 (26.7%)
Tuberculum sellae	2 (6.7%)
Cerebellopontine angle	3 (10.0%)
Petroclival	3 (10.0%)
Tentorial	3 (10.0%)
High cervical level	1 (3.3%)
Torcular	1 (3.3%)
Orbital	1 (3.3%)
WHO grade, *n* (%)	
I	18 (60.0%)
II	1 (3.3%)
III	0 (0%)
Unknown	11 (36.7%)
Surgical resection before SRT, *n* (%)	
Yes	18 (60.0%)
No	12 (40.0%)

*MET-PET* ^11^C-Methionine positron emission tomography, *MRI* magnetic resonance imaging, *SRT* stereotactic radiotherapy, *WHO* World Health Organization

**Table 2:** Characteristics of patients with PD

	Original histopathology before SRT	Tumor location	Treatment after PD (months after SRT)
Case 7	Meningothelial meningioma WHO grade 1	Sphenoidal	1st recurrence (61 months): re-resection (PA: meningothelial meningioma WHO grade 1)
Case 18	Meningioma WHO grade 1 NOS	Sphenoidal	1st recurrence (57 months): re-resection (PA: atypical meningioma WHO grade 2) 2nd recurrence (97 months): reirradiation
Case 27	Meningothelial meningioma WHO grade 1	Parasagittal	1st recurrence (31 months): reirradiation 2nd recurrence (62 months): BSC/palliative
Case 34	Fibrous meningioma WHO grade 1	Parasagittal	1st recurrence (24 months): reirradiation

*PD* progressive disease, *SRT* stereotactic radiotherapy, *PA* pathology result, *NOS* not otherwise specified, *BSC* best supportive care

### Pre- and post-SRT ^11^C-Methionine uptake

The comparison of pre-SRT and post-SRT MET-PETs is shown in Table 3. Both visually and quantitatively, the uptake of ^11^C-Methionine remained strongly elevated after SRT. In 15 patients the pre-SRT ^11^C-Methionine T/N-ratios could be calculated. Median values (IQR) were SUV_max_ T/N_R front_ 2.80 (2.07–3.33), SUV_max_ T/N_mirror_ 2.98 (2.29–3.34), SUV_peak_ T/N_R front_ 2.47 (1.91–3.52) and SUV_peak_ T/N_mirror_ 2.40 (2.01–3.49). The median (IQR) values for the 47 post-SRT MET-PETs were SUV_max_ T/N_R front_ 2.80 (2.10–3.66), SUV_max_ T/N_mirror_ 2.92 (2.18–3.68), SUV_peak_ T/N_R front_ 2.33 (1.62–3.42) and SUV_peak_ T/N_mirror_ 2.38 (1.90–3.41). There was no significant difference between the pre-SRT and post-SRT MET-PET. Also, no significant difference could be detected in comparing the subgroups of MET-PETs of patients with SD and PD to the pre-SRT MET-PETs. Effect sizes were small to very small.

**Table 3:** Comparison pre-SRT MET-PET scans (*N* = 15) vs. post-SRT MET-PET scans (*N* = 47)

		p*	*r*
**SUV**_**max**_** T/N**_**R front**_			
Median pre-SRT (IQR): 2.80 (2.07–3.33)	Median post-SRT-*all-*(IQR): 2.80 (2.10–3.66)	0.79	− 0.03
	Median post-SRT-*stable disease-*(IQR): 2.65 (2.13–3.68)	0.76	− 0.04
	Median post-SRT-*progressive disease-*(IQR): 2.97 (1.55–3.54)	1	0
**SUV**_**max**_** T/N**_**mirror**_			
Median pre-SRT (IQR): 2.98 (2.29–3.34)	Median post-SRT-*all-*(IQR): 2.92 (2.18–3.68)	0.98	0
	Median post-SRT-*stable disease-*(IQR): 2.92 (2.19–3.71)	0.95	− 0.09
	Median post-SRT-*progressive disease-*(IQR): 2.95 (1.74–3.60)	0.7	− 0.08
**SUV**_**peak**_** T/N**_**R front**_			
Median pre-SRT (IQR): 2.47 (1.91–3.52)	Median post-SRT*-all-*(IQR): 2.33 (1.62–3.42)	0.43	− 0.10
	Median post-SRT*-stable disease-*(IQR): 2.35 (1.64–3.40)	0.47	− 0.10
	Median post-SRT*-progressive disease-*(IQR): 2.25 (1.44–3.74)	0.48	− 0.15
**SUV**_**peak**_** T/N**_**mirror**_			
Median pre-SRT (IQR): 2.40 (2.01–3.49)	Median post-SRT*-all-*(IQR): 2.38 (1.90–3.41)	0.51	− 0.08
	Median post-SRT*-stable disease-*(IQR): 2.38 (1.91–3.36)	0.52	− 0.09
	Median post-SRT*-progressive disease-*(IQR): 2.35 (1.56–3.72)	0.64	− 0.10

*SRT* stereotactic radiotherapy, *MET-PET* ^11^C-Methionine positron emission tomography, *IQR* interquartile range, *T/N* tumor-to-normal ratio, *r* effect size

### ^11^C-Methionine uptake in patients with stable vs. progressive disease

Twenty-five patients (with 26 SRT treated meningiomas) had stable disease (41 SD FU MET-PET scans) versus 4 patients with progressive disease (6 PD FU MET-PET scans). There was no significant difference in uptake between MET-PET scans with stable versus progressive disease in this series, with median (IQR) SUV_max_ T/N_R front_ for SD and PD (*p*) 2.65 (2.13–3.68) and 2.97 (1.55–3.54) [*p* = 0.66];_,_ SUV_max_ T/N_mirror_ 2.92 (2.19–3.71) and 2.95 (1.74–3.60) [*p* = 0.61]; SUV_peak_ T/N_R front_ 2.35 (1.64–3.40) and 2.25 (1.44–3.74) [*p* = 0.80]; SUV_peak_ T/N_mirror_ 2.38 (1.91–3.36) and 2.35 (1.56–3.72) [*p* = 0.95] (Table 4). Effect sizes were small to very small.

**Table 4:** Comparison stable disease MET-PET scans (*n* = 41) vs. progressive disease MET-PET scans (*n* = 6)

	Stable disease median (IQR)	Progressive disease median (IQR)	p*	*r*
SUV_max_ T/N_R front_	2.65 (2.13–3.68)	2.97 (1.55–3.54)	0.66	− 0.07
SUV_max_ T/N_mirror_	2.92 (2.19–3.71)	2.95 (1.74–3.60)	0.61	− 0.07
SUV_peak_ T/N_R front_	2.35 (1.64–3.40)	2.25 (1.44–3.74)	0.80	− 0.04
SUV_peak_ T/N_mirror_	2.38 (1.91–3.36)	2.35 (1.56–3.72)	0.95	− 0.01

*MET-PET*1 1C-methionine positron emission tomography, *IQR* interquartile range, *SUV* standardized uptake value, *T/N* tumor-to-normal ratio, *r* effect size

### Longitudinal ^11^C-Methionine uptake after stereotactic radiotherapy

**Fig. 2:**
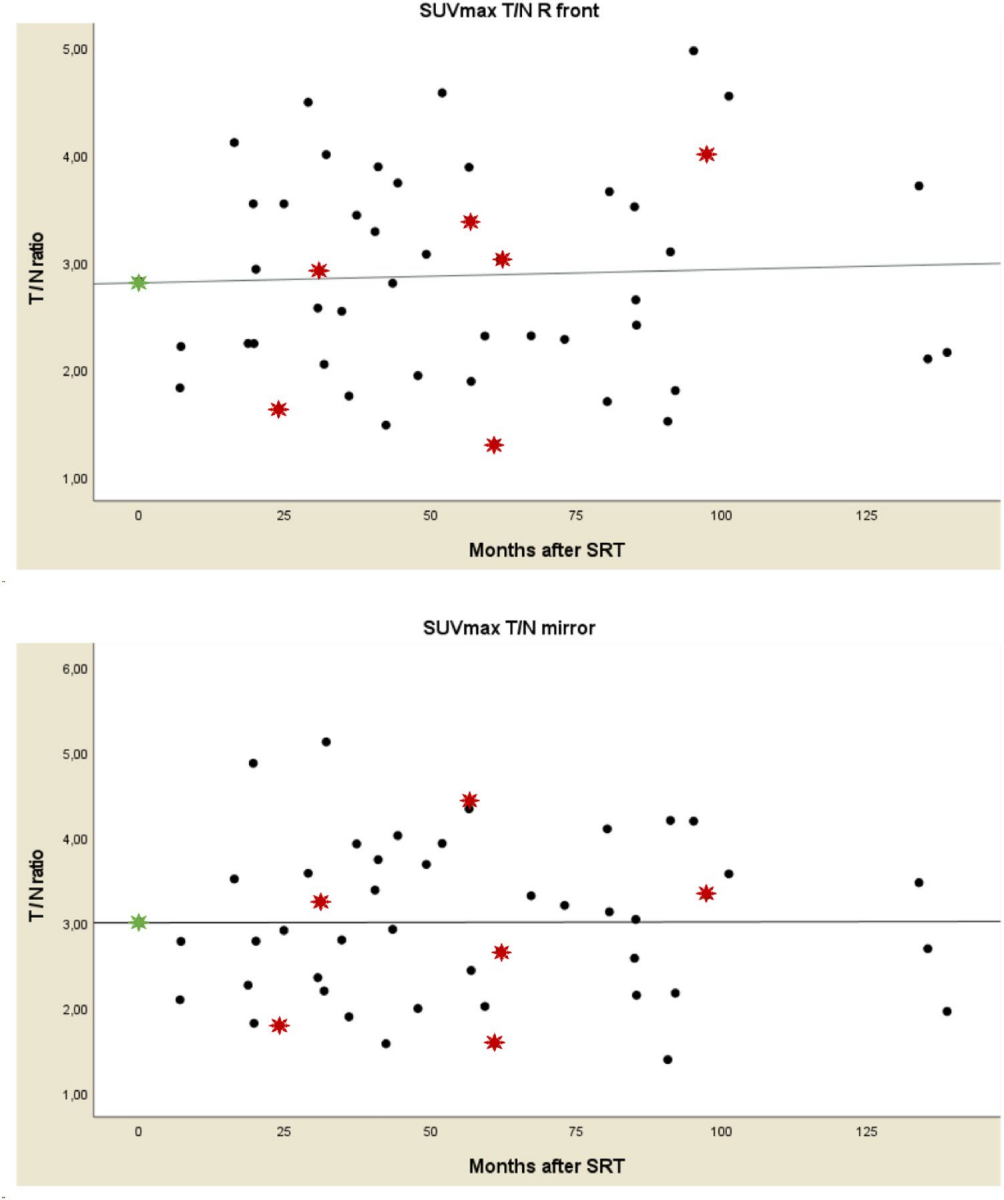

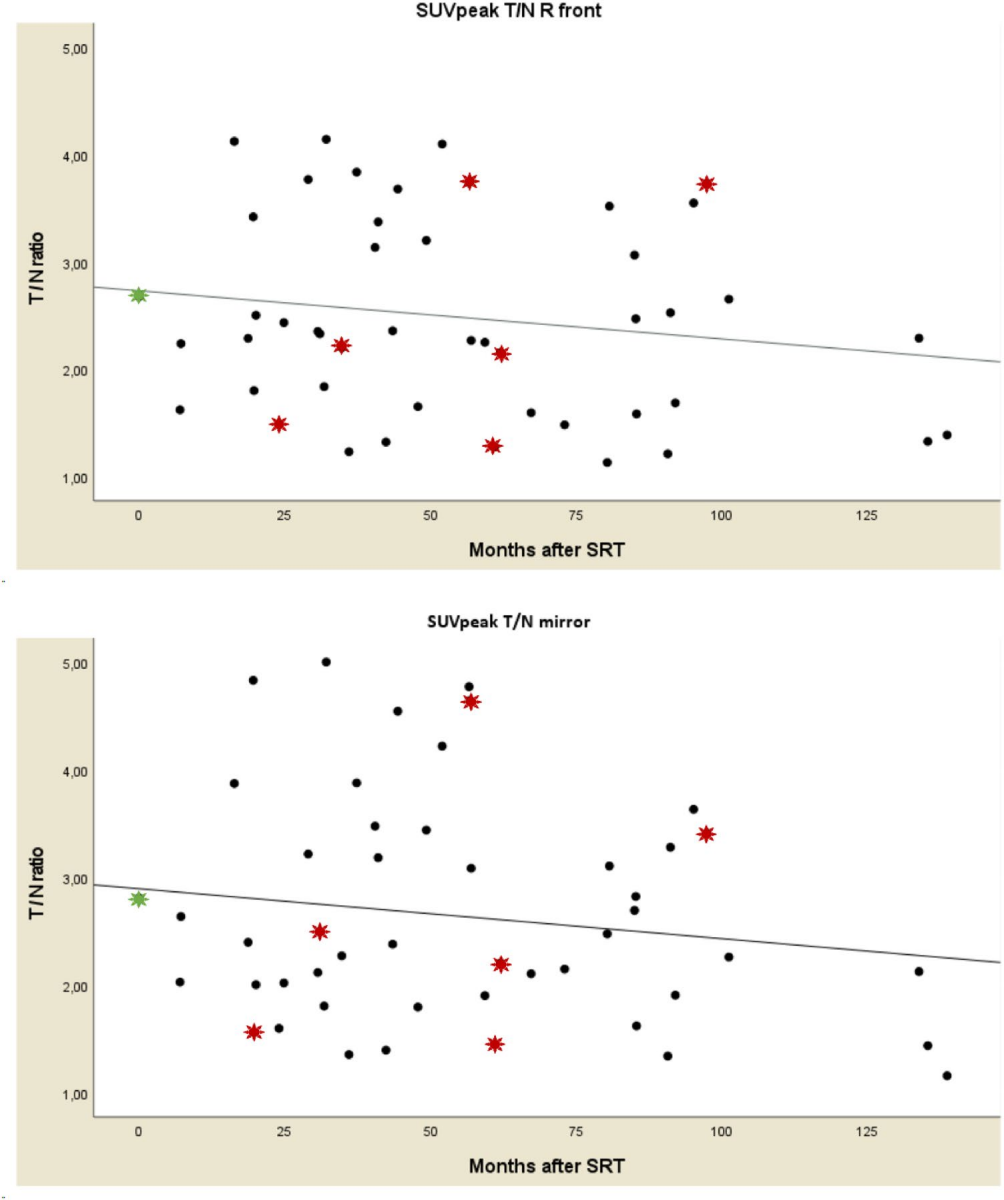
Scatterplots of T/N-ratios showing the uptake ratios for the patients with SD and PD over time. Black filled circle = MET-PET T/N ratio of patients with SD. Red filled star = MET-PET T/N ratio of patients with PD. Green filled star = mean value pre-SRT MET-PET T/N ratio. *T/N* tumor-to-normal, *SD* stable disease, *PD* progressive disease, *SUV* standardized uptake value, *T/N* tumor-to-normal ratio, *SRT* stereotactic radiotherapy

**Fig. 3:**
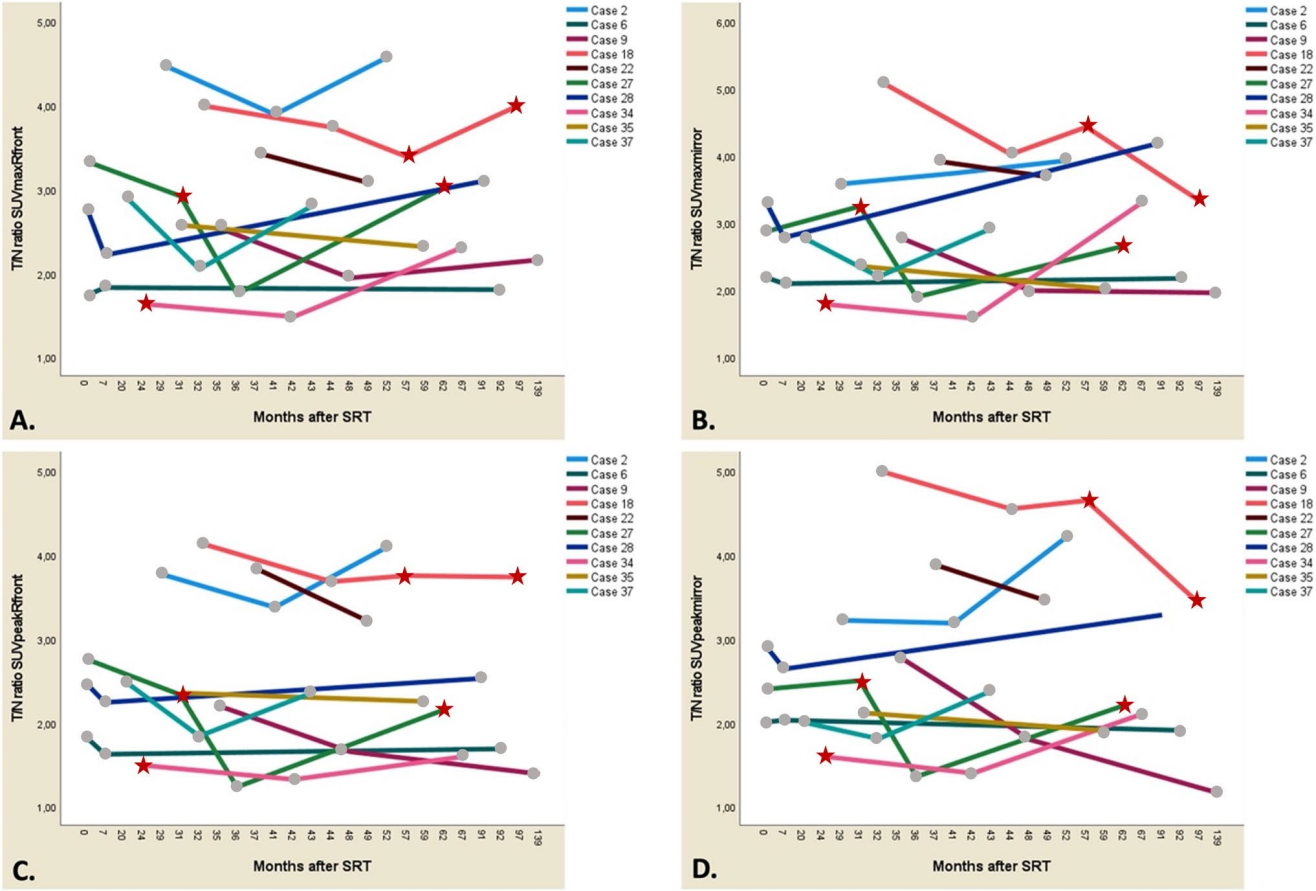
Uptake patterns for the four different T/N-ratios (**A** SUV_max_ T/N_R front_, **B** SUV_max_ T/N_mirror,_ **C** SUV_peak_ T/N_R Front_, **D** SUV_peak_ T/N_mirror_) of the 10 tumor cases which had a long follow-up duration of > 3.5 years and at least 2 or more MET-PETs during follow-up. Red filled star = PD MET-PETs, Ash filled circle = SD MET-PETs. Tumor cases 18, 27 and 34 experienced PD during follow-up, on respectively 57 and 97 months after SRT (case 18), 31 and 62 months after SRT (case 27) and 24 months after SRT (case 34). Tumor cases 2 and 37 are two meningiomas within the same patient, treated with SRT on a different timepoint. *MET-PET* ^11^C-Methionine positron emission tomography, *T/N* tumor-to-normal, *SUV* standardized uptake value, *SRT* stereotactic radiotherapy

Scatterplots of the four T/N-ratios were created (Fig. 2), displaying uptake during FU. Patients with PD are marked as red dots. The plots show that MET-PET T/N-ratios are very dispersed. No significant correlation during FU was found (Spearman’s ρ correlation coefficient 0.04 for SUV_max_ T/N_R front_ [*p* = 0.79]; 0.06 for SUV_max_ T/N_mirror_ [*p* = 0.70]; -0.17 for SUV_peak_ T/N_R front_ [*p* = 0.24]; − 0.13 for SUV_peak_ T/N_mirror_ [*p* = 0.39]). All T/N-ratios remained elevated > 1.00. For 15 patients, the pre-SRT T/N ratio could be compared to the post-SRT T/N ratio; the percental and absolute ∆ SUV T/N are given (Table 5)*.* Ten patients with meningiomas had a FU duration longer than 3.5 years and at least 2 MET-PETs during FU. Individualized uptake patterns of these patients are shown in Fig. 3*.* Three of these cases were found to have progressive disease. MET-PET scans of patients with PD are marked with red asterisks. The MET-PET scans at the moment of PD do not consistently show a higher ^11^C-Methionine uptake compared to SD MET-PET scans. Neither is there a consistent rise in ^11^C-Methionine uptake at the moment of PD. Also, quite some patients with SD show a rise in ^11^C-Methionine accumulation during FU, without a clinical correlate.

**Table 5:** Percental and absolute SUV T/N ratio difference for the 15 patients of which a pre-SRT MET-PET could be compared to post-SRT MET-PETs

Type of T/N ratio	Mean (SD) percental ∆ pre-SRT T/N ratio − post-SRT T/N ratio	Mean (SD) absolute ∆ pre-SRT T/N ratio − post-SRT T/N ratio
SUV_max_ T/N_R front_	− 3.62% (23.62)	− 0.21 (0.63)
SUV_max_ T/N_mirror_	− 2.19% (38.85)	− 0.19 (0.86)
SUV_peak_ T/N_R front_	− 15.43% (14.39)	− 0.42 (0.41)
SUV_peak_ T/N_mirror_	− 8.15% (25.10)	− 0.30 (0.52)

The median follow-up duration (range) in this subgroup was 61.8 months (7.1–91.5)

*T/N* tumor-to-normal, *SRT* stereotactic radiotherapy, *MET-PET* ^11^C-Methionine positron emission tomography, *SD* standard deviation, *SUV* standardized uptake value

## Discussion

For various reasons, amino acid PET would seem to be the perfect add-on to MRI in meningioma FU, especially if the FU MRI of complex meningiomas is difficult to interpret in the setting after SRT. Unfortunately, our data show that the ^11^C-Methionine uptake remains elevated, even many years after SRT. We did not find a robust decrease in neither patients with SD nor with PD. Also, in the (small) group of patients with PD, no significant increase was encountered. No intergroup difference between patients with SD and PD was detected. Therefore, we could not find any benefit of MET-PET in our institutional patient series of complex meningioma patients after SRT.

In the literature there currently is a paucity of data to explain why the T/N uptake ratio of ^11^C-Methionine remains elevated in meningiomas after SRT. How radiation interacts with cellular methionine metabolism is not yet completely understood [33]. Earlier studies found that radiotherapy reduces methionine levels when tissue is damaged (e.g., day 6 after radiotherapy), but that methionine levels recover to pre-radiotherapy levels upon tissue recovery [33]. This could play a role in the findings of our study. Also the pathophysiological mechanisms involved in radio-induced cerebral damage are incompletely understood. Historically, this was thought to be solely related to DNA damage of brain-glial cells and vascular-endothelial cells. But modern literature also describes an inflammatory response as response of neural tissue to irradiation [34]. Since reactive inflammation can lead to MET accumulation, it is not impossible that this may play a role [3]. However, the exact time path of radiotherapy induced inflammation remains to be elucidated. Theoretically other mechanisms could play a role. SRT is capable of disrupting the blood–brain barrier, which could be an explanation for leaking of ^11^C-Methionine tracer after SRT. But, since meningiomas are extra-axial tumors located outside the blood–brain barrier, this is probably not an explanation for the ^11^C-Methionine accumulation encountered in this tumor type. Disruption of the blood–brain barrier is more likely to play a role in ^11^C-Methionine uptake in intra-axial lesions, like for example gliomas, after radiotherapy. More advanced MET-PET analysis methods, e.g., like quantitative kinetic analysis using a compartiment model, could be a useful research strategy to further explore the mechanism behind ^11^C-Methionine accumulation in meningiomas after SRT in future studies.

Other types of amino acid PET are available of which ^18^F-Fluoroethyl-l-tyrosine PET (FET-PET) is the most prevalent. Where MET-PET necessitates an onsite cyclotron, FET-PET overcomes this practical problem, having a longer half-life. FET-PET can be performed in hospitals without a cyclotron and is therefore more widespread available. For this reason FET-PET gained popularity [6], which explains its application in many neuro-oncological studies [9, 10, 13, 20, 35]. A very large German PET-center, performing more than 6500 FET-PETs, recently published their indications for FET-PET imaging [36]. Remarkably, they reported that FET-PET was mainly used to detect glioma recurrence and metastatic recurrence. Meningioma FU was not mentioned as an indication for FET-PET in their extensive series. This could be due to the persistent high uptake of amino acid tracer, which makes interpretation of PET results difficult.

It is noteworthy to mention that most current PET-studies involving meningiomas focus on the value of PET tracers targeted to the somatostatin receptor 2 (SSTR-2), like ^68^Ga-DOTATOC PET and ^68^Ga-DOTATE PET. This technique has been used for meningioma detection [37–41], tumor prognosis [42], treatment planning [39, 43–46], and during FU [40, 47–49]. The abundancy of these publications touch upon tumor diagnostics and tumor-target delineation for SRT [37–41, 43, 44, 46]. Fewer studies investigate the role of ^68^Ga-DOTATOC and ^68^Ga-DOTATATE PET in FU and evaluation of treatment response [39, 40, 48, 49]. A recent example of such a study is the publication of Lütgendorf-Caucig et al. on ^68^Ga-DOTATOC PET during FU of benign meningioma after proton therapy [49]. The authors report an early trend of SUV-decrease in their 22 patients but noticed some ambiguous outcomes on other study parameters. Their series included no PD-patients. They conclude that further translational research is needed to understand the underlying biology. Barone et al. published a series of 12 meningioma patients with a ^68^Ga-DOTATOC PET after gamma knife radiosurgery and report a decrease of uptake in seven patients, a stable uptake in two patients, and an increase of uptake in three patients. Kowalski et al. found a decrease of 36% for SUV_max_ and 14.7% for SUV_mean_ of ^68^Ga-DOTATATE PET uptake in a subgroup of ten meningioma patients, 3 months after radiotherapy and compared to the pre-radiotherapy values, with a very wide range in their outcomes. Last, Ivanidze et al. conclude that they can achieve an excellent differentiation between meningioma and post-treatment change, based on their approach using target lesion/superior sagittal sinus SUV_max_ ratios. Due to a paucity of literature supporting the role of PET with SSTR2 targeted tracers in meningioma FU, future studies will have to prove its definitive value. However, with a cell-surface receptor as the target, this SSTR2 targeted PET is theoretically expected to be less informative on the metabolic state of a tumor, which might be a limitation in the detection of tumor proliferation.

In the 2017 report of the RANO/PET group on PET in meningioma patients during FU, it is concluded that there is only preliminary (level III) evidence for a role of amino acid PET in treatment monitoring after radiotherapy. Regarding ^68^Ga-DOTATOC PET, there is level II evidence for differentiation between tumor progression and post-therapeutic reactive changes [24]. Unfortunately, our institutional experience could not add further support to the role of MET-PET for FU purpose in irradiated meningioma patients.

### Limitations

The retrospective nature of this study might potentially have led to a selection bias. The decision to perform FU MET-PET and the timing of the investigation was at the discretion of the treating physician. There was no protocol with fixed time-intervals for FU MET-PET imaging, leading to a substantial spread in the interval between SRT and the MET-PET scans. Also, due to changes in saving protocols, a substantial amount of older MET-PETs was not available for quantitative analysis. In this study, it was chosen to analyze SUV_max_ and SUV_peak_. SUV_mean_ as other more advanced parameters were not part of the study protocol. Last, the current study might be considered underpowered. Ideally, a higher number of included patients in our study would have been better, leading to more patients with SD and PD. But because of the rarity of this type of disease and institutional differences in acquiring and analyzing MET-PET, it is unlikely that such series will ever appear. Moreover, in our data, we did not find the slightest trend towards a significant decrease in uptake or any difference between patients with SD versus PD. In this scientific field, most studies have low number of patients and the current series is one of the largest available on the topic of amino acid PET after SRT in complex meningioma patients.

## Conclusion

The current study evaluates one of the largest series of ^11^C-Methionine uptake in skullbase meningiomas or meningiomas in close relation to vascular structures after SRT. Our results show a remarkably persistent elevated-^11^C-Methionine uptake in meningiomas after SRT, with no significant difference between patients with stable versus progressive disease during FU. Therefore, our data do not support the use of MET-PET during the FU of patients with a complex meningioma after SRT.

### Supplementary Information

Below is the link to the electronic supplementary material.

Supplementary file1 (DOCX 575 kb)

## Data Availability

Raw data of this study is available upon reasonable request by way of contacting the corresponding author.

## Abbreviations

FDG-PET: ^18^F-Fluorodeoxyglucose positron emission tomography
FET-PET18: F-Fluoroethyl-l-tyrosine positron emission tomography
FU: Follow-up
LINAC: Linear accelerator
MET-PET: ^11^C-Methionine positron emission tomography
MRI: Magnetic resonance imaging
PD: Progressive disease
PET: Positron emission tomography
ROC: Receiver-operating-characteristic
ROI: Region of interest
SD: Stable disease
SRT: Stereotactic radiotherapy
SSTR2: Somatostatin receptor 2
SUV: Standardized uptake value
T/N: Tumor-to-normal
