# Supplementary material for: 11C-Methionine uptake in meningiomas after stereotactic radiotherapy
Source: Ann Nucl Med. 2024 May 8;38(8):596–606. doi: 10.1007/s12149-024-01932-6 (PMC11282149; doi:10.1007/s12149-024-01932-6)
Supplement: Supplementary file 1 — Supplementary file1 (DOCX 575 kb) [file 12149_2024_1932_MOESM1_ESM.docx]

**Supplementary data**

The visualization illustrates how SUV measurements were performed in this study.


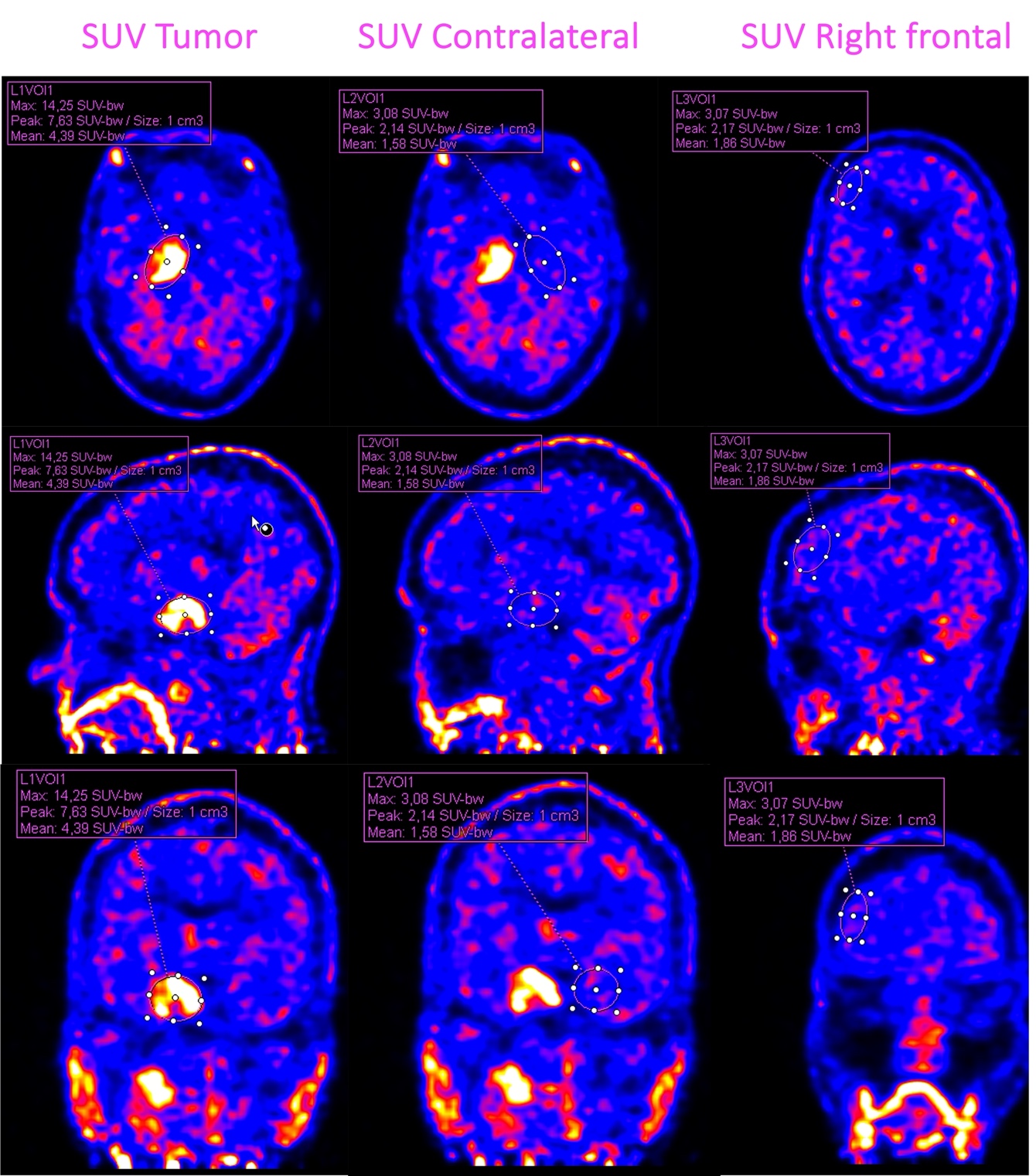


Abbreviations: SUV = Standardized uptake value
